# Risk factors analysis and research on the construction of early prediction model of difficult weaning in children with mechanical ventilation

**DOI:** 10.3389/fped.2025.1630580

**Published:** 2025-08-01

**Authors:** Wenlan Zhang, Hua Lu, Xiaoliao Tang, Suqin Xia, Jian Zhang, Jiwen Sun, Nanping Shen, Hong Ren

**Affiliations:** ^1^ PICU, Shanghai Children’s Medical Center, Shanghai Jiao Tong University School of Medicine, Shanghai, China; ^2^ PICU, Fujian Children’s Hospital (Fujian Branch of Shanghai Children’s Medical Center), College of Clinical Medicine for Obstetrics & Gynecology and Pediatrics, Fujian Medical University, Fujian, China; ^3^ Department of Nursing, Shanghai Children’s Medical Center, Shanghai Jiao Tong University School of Medicine, Shanghai, China

**Keywords:** mechanical ventilation, ultrasonics, ventilator weaning, pediatric intensive care unit, forecasting

## Abstract

**Objective:**

To identify risk factors for difficult weaning in mechanically ventilated children and develop an early predictive nomogram.

**Methods:**

A prospective observational study was cunducted between Aug/2023 and Nov/2024 involving 205 pediatric patients from two PICUs. General demographic and clinical data were collected, along with lung ultrasound (LUS) scores obtained within 48–72 h of initiating mechanical ventilation. Additional respiratory and oxygenation function-related parameters were also synchronously recorded. All pediatric patients were followed up to their weaning outcomes, duration of mechanical ventilation, and ICU stay days.Weaning outcomes were defined as the dependent variable, while the collected clinical indicators were treated as independent variables for univariate analysis. Multivariable logistic regression analysis was performed to identify significant predictors, and a nomogram was developed and validated using ROC and K-S curves.

**Results:**

This study included 205 mechanically ventilated pediatric patients with complete data, and the incidence of difficult weaning was 47.8%. Two independent risk factors were identified: lung ultrasound (LUS) score (OR = 2.316, 95% CI: 1.668–3.216, *P* < 0.001) and pediatric critical illness score (PCIS) (OR = 0.748, 95% CI: 0.639–0.875, *P* = 0.001). The nomogram demonstrated good discriminatory ability, with an AUC of 0.874 in the modeling cohort and 0.854 in the validation cohort.

**Conclusion:**

LUS scores and PCIS are significant early predictors of difficult weaning in mechanically ventilated pediatric patients. The validated nomogram offers a reliable tool for quantitative risk stratification, which can support the development of personalized ventilation liberation strategies.

## 1 Introduction

The American clinical practice guidelines for Mechanical Ventilation Liberation in Critically Ill Patients conceptualize ventilator liberation as a multidimensional process encompassing both the terminal phase of endotracheal tube removal (extubation) and the antecedent structured weaning protocol (1). This comprehensive framework integrates systematic patient evaluation, targeted therapeutic interventions, and specialized nursing care to optimize readiness for successful ventilator discontinuation. Clinical researchs reveals that approximately 45% of pediatric patients experience protracted weaning duration (designated as weaning difficulty) (2), frequently culminating in extubation failure, extended intensive care unit (ICU) length of stay, and elevated mortality rates (3, 4). Accordingly, the guidelines advocate for systematic assessment of patients requiring mechanical ventilation beyond 48 h to evaluate early weaning feasibility, thereby mitigating risks associated with ventilator-associated complications (1).

Contemporary epidemiological studies demonstrate persistent high incidence rates of pediatric weaning difficulty, fostering prevalent clinical reliance on empirical judgment and conservative management approaches. This practice pattern predisposes to either delayed weaning initiation or suboptimal recognition of weaning readiness indicators, while prolonged invasive mechanical ventilation paradoxically exacerbates weaning challenges (5, 6). Bedside lung ultrasound is a widely utilized non-invasive tool in the intensive care unit (ICU) for effectively assessing pulmonary function and guiding clinical decision-making (7–9). Moreover, the severity of illness has been shown to be significantly associated with weaning outcomes in pediatric patients. However, there is currently a lack of integrated predictive models available for the early identification of difficult weaning in children.

This investigation addresses the challenge of inadequate risk assessment for difficult weaning in pediatric populations. Through systematic examination and analyzing risk factors throughout the weaning process and incorporating lung ultrasound (10), we developed and validated an early predictive model for adverse weaning outcomes and detecting the modifiable risk profiles to guide to establishing an evidence-based framework for personalized pulmonary rehabilitation strategies and enhance weaning success rates.

## 2 Materials and methods

### 2.1 Study population

This prospective multicenter cohort study employed consecutive convenience sampling across two tertiary pediatric referral centers in Shanghai, China, and Fujian Province, with enrollment spanning August 1, 2023 through November30, 2024. The study population comprised pediatric patients requiring invasive mechanical ventilation via endotracheal intubation in pediatric intensive care units (PICUs).

#### 2.1.1 Inclusion criteria

Duration of invasive mechanical ventilation exceeding 48 h and whose age ranged between 29 days and 18 years. This age range was selected to account for developmental variations in respiratory physiology, while conforming to World Health Organization (WHO) pediatric definitions and aligning with previous cohort studies.

#### 2.1.2 Exclusion criteria

1. Chronic tracheostomy dependence
2. Structural airway anomalies (congenital/acquired stenosis, malacia)
3. Thoracic trauma impairing sonographic window acquisition
4. Neuromuscular disorders affecting respiratory drive (e.g., spinal muscular atrophy, myasthenic syndromes)
5. Pre-existing ventilator dependence prior to PICU admission
6. Cyanotic congenital heart disease (unrepaired/palliated)
7. End-stage oncological diagnoses with anticipated <6-month survival
8. Mortality events during mechanical ventilation phase
9. Unplanned extubation episodes requiring emergent reintubation

### 2.2 Study design and settings

This study was conducted by the Pediatric Intensive Care Unit (PICU) at Shanghai Children's Medical Center, a tertiary pediatric care institution affiliated with Shanghai Jiao Tong University School of Medicine, in collaboration with its affiliated branch clinical sites in Fujian Province. The two institutions share identical patient admission procedures, as well as comparable levels of medical care and management frameworks. The multidisciplinary research team comprised eight credentialed professionals, including principal investigators, PICU physicians, critical care ultrasonography nurses, and licensed respiratory therapists, all possessing specialized expertise in ventilator weaning protocols. Ethical compliance was ensured through formal review and approval by the Institutional Review Board (IRB) of the host institution (Ethics Approval Nos.: 2022ETKLRKLRK08001; SCMCIRB-YN2022007), adhering to the Declaration of Helsinki principles. Legally authorized guardians of all enrolled participants provided written informed consent following comprehensive protocol disclosure.

Both pediatric intensive care units (PICUs) adhered to identical ventilator liberation protocols that were consistent with established clinical practice guidelines. The study constitutes a prospective observational cohort design, developed in accordance with recommended standards for ventilator liberation research (10–12). Standardized training on LUS scoring and weaning assessment was conducted for all staff prior to study initiation, ensuring inter-institutional consistency in patient management. LUS examinations were performed by 4 certified intensivists who completed ≥50 supervised scans. Inter-rater reliability was confirmed (Cohen's Kappa = 0.853) through blinded re-evaluation of 50% randomly selected scans.

Protocolized daily readiness assessments were systematically implemented for pediatric patients requiring mechanical ventilation beyond 48 h to evaluate their eligibility for weaning initiation. A standardized lung ultrasonography scoring tool (LUS) was utilized to assess the extent of lung injury within 48–72 h post-intubation among eligible participants. Concurrently, comprehensive respiratory and oxygenation function-related indicators were systematically collected within one hour before or after the administration of lung ultrasound. The weaning outcomes of the patients were longitudinally monitored using the WIND (Weaning According to a New Definition) classification framework developed by the European Mechanical Ventilation Research Collaborative Group (6). Subsequently, patients were categorized into two groups: the simple weaning group and the difficult weaning group. The endpoint of the study was defined as either discharge from the PICU or death, whichever occurred first. This classification standard is also applicable to pediatric populations (3).

The primary outcomes focused on the incidence of difficult weaning in mechanically ventilated children and the identification of risk factors associated with difficult weaning. Using statistical software, 70% of the case data (modeling group) was randomly selected from the overall included sample to construct a predictive model, while the remaining 30% of the sample (validation group) was used to test and evaluate the predictive performance of the model.

### 2.3 Definitions and implementation framework

According to the WIND criteria established by the European Collaborative Group on Mechanical Ventilation Research, the ventilator liberation process is operationally defined as the interval commencing with the initial spontaneous breathing trial (SBT) and culminating in successful endotracheal extubation with complete ventilator independence (3, 6). Patients’ weaning outcomes are stratified based on the results of the first SBT. Simple weaning is defined as successful extubation within 24 h following the successful completion of the first SBT. Difficult weaning is characterized by either failure of the initial SBT requiring ≥2 subsequent SBT attempts to achieve extubation readiness or an inability to successfully extubate within 24 h despite passing the first SBT (6).

This investigation strictly adheres to internationally recognized ventilator liberation guidelines and multidisciplinary expert consensus protocols (5, 12). Structured pre-spontaneous breathing trial (SBT) evaluations were systematically performed to validate weaning eligibility across all pediatric cases. Daily SBT readiness assessments were triggered when:

(1) Patients were required to meet the following clinical criteria: effective recovery of the primary etiology underlying respiratory failure; normal and stable hemodynamic status or stability maintained with vasoactive drug support-defined as the administration of either no vasopressors or only low-dose vasopressors (e.g., dopamine ≤5 μg/kg/min, norepinephrine <0.2 μg/kg/min, or equivalent agents, along with no escalation in vasoactive agent use over the preceding 24 h and absence of clinical signs of shock; sustained and effective spontaneous breathing in SIMV (synchronized intermittent mandatory ventilation) mode (respiratory rate gradually reduced to one-half to two-thirds of the original control ventilation rate, FiO_2_ ≤ 50%, PEEP ≤ 8 cmH_2_O. The target physiologic tidal volume (Vt) should be maintained at 5–8 ml/kg while avoiding Vt >10 ml/kg of ideal body weight); intact airway protective reflexes, absence of multiorgan dysfunction syndrome, and no significant metabolic disturbances(pH > 7.25, absence of hypercapnic respiratory failure, and stable metabolic status, e.g., normoglycemia, euvolemia, normal electrolyte levels) (1, 5, 11, 12).
(2) The common ventilatory parameter optimization was performed with the following requirements: FiO_2_ < 50%, PEEP (positive end-expiratory pressure) ≤5–8 cmH_2_O, PIP(peak inspiratory pressure) <15 cmH_2_O; an unobstructed airway, respiratory rate <30–35 breaths/min, tidal volume ≥5 ml/kg, SpO_2_ ≥ 94% (or PaO_2_ > 60 mmHg), PaCO_2 _< 50 mmHg, and P/F ratio(oxygenation index) >150 (1, 4, 5, 11, 12).
(3) Exclusion criteria included sustained tachyarrhythmias, refractory shock states, and neurological compromise (Glasgow Coma Scale ≤8), RASS(Richmond Agitation-Sedation Scale) ≤−3 (11–13).

Following the confirmation of SBT eligibility, a 120 min SBT is conducted using the CPAP (continuous positive airway pressure) ventilation mode (12, 13). Simultaneously, diaphragmatic function is assessed via bedside ultrasonography, and PiMax (maximum inspiratory pressure) is continuously monitored to exclude severe respiratory muscle dysfunction (12). According to established standards (5, 11–13), SBT success is defined as the absence of any clinical signs of respiratory distress during the SBT process, including dyspnea, nasal flaring, paradoxical breathing, apnea, heart rate variability exceeding 20% above baseline, a decrease in SpO_2_ (peripheral oxygen saturation) >5%, very weak diaphragmatic activity, PiMax persistently below 20 cmH_2_O, an elevation in PaCO_2_ > 10 mmHg, or a P/F ratio <150. Conversely, SBT failure is designated upon the presence of any of the aforementioned criteria. Extubation failure is defined as the need for reintubation within 48 h after decannulation due to the occurrence of severe respiratory distress or respiratory failure (11, 12).

### 2.4 Data collection

Through a review of literature published over the past decade, followed by a structured screening and team discussion process, we identified key factors and clinical indicators associated with difficult weaning in pediatric patients and established the following parameters:

#### 2.4.1 Demographic and clinical data collection

Upon admission to the pediatric intensive care unit (PICU), systematic documentation of clinical baseline parameters was performed, including age, gender, primary diagnosis, and PCIS (Pediatric Critical Illness Score), a validated assessment tool developed by the Emergency Medicine Committee of the Pediatric Society, Chinese Medical Association (14). Nutritional status was objectively evaluated using anthropometric indices, specifically weight-for-age Z-scores (WAZ), calculated with WHO-designated software (Anthro v3.2.2 and AnthroPlus v1.0.4) and standardized growth references. Malnutrition was operationally defined as WAZ < −2 SD (15, 16). Pharmacological interventions, including the administration of vasoactive agents (e.g., epinephrine, norepinephrine) or neuromuscular blocking agents, were prospectively recorded. Longitudinal tracking of mechanical ventilation duration (ventilator-free days) and ICU length of stay (LOS) was maintained.

#### 2.4.2 Lung ultrasound score (LUS)

A standardized pulmonary ultrasonography protocol with semi-quantitative assessment was implemented within 48–72 h following mechanical ventilation initiation in pediatric patients. Systematic bilateral lung evaluations were performed across 12 predefined thoracic regions (7–10). Sonographic patterns reflecting pulmonary aeration deficits were stratified into four severity grades based on validated diagnostic criteria. For each anatomical region, the most severe aeration abnormality was identified and assigned a weighted score (0–3) according to standardized grading matrices (Table 1) (10, 17, 18). The cumulative LUS was calculated by summing scores from all 12 regions (range: 0–36), serving as a quantitative measure of global pulmonary aeration efficiency. Elevated LUS values inversely correlate with functional residual capacity and directly associate with oxygenation impairment severity, indicative of reduced alveolar recruitment and progressive pulmonary dysfunction (10, 17, 18).

**Table 1:** Standardized sonographic scoring system for pulmonary aeration loss.

Pattern classification	Degree of aeration	Sonographic features	Assigned score
N	Normal aeration	Presence of lung sliding with A-lines or ≤2 B-lines	0 points
B1	Moderate aeration loss	3–6 discrete B-lines within a single scanning field	1 point
B3	Severe aeration loss	≥6 B-lines or coalescent B-line patterns within a single scanning field	2 points
C/P	Extremely severe aeration loss	Presence of any of the following signs within a single scanning field: Tissue-like sign (alveolar consolidation), Fragmentation sign (subpleural atelectasis), Pleural effusion patterns: Jellyfish sign, Quadrilateral sign	3 points

#### 2.4.3 Respiratory and oxygenation function monitoring parameters

Within one hour preceding or following pulmonary ultrasonography, synchronized arterial blood gas (ABG) analysis was performed alongside continuous hemodynamic and oxygenation monitoring. All measurements were standardized to reduce procedural variability, with FiO2 levels recorded at the time of ABG sampling and the most recent same-day blood biochemical analysis.

#### 2.4.4 Key parameters included

1. ABG Analysis: pH, arterial oxygen tension (PaO_2_), arterial carbon dioxide tension (PaCO_2_), oxyhemoglobin saturation (SaO_2_), lactate (Lac) levels.
2. Calculated Metrics: Oxygenation index (P/F ratio = PaO_2_/FiO_2_).
3. Physiological Monitoring: heart rate (HR), regional cerebral oxygen saturation (rSO_2_) via near-infrared spectroscopy (NIRS).
4. Richmond Agitation-Sedation Scale (RASS) scores to quantify sedation-agitation equilibrium.
5. Blood Biochemical Analysis: serum albumin(ALB), hemoglobin concentration (HB).

Data collection was conducted by certified researchers from the PICU Liberation working group after completing protocol-driven training programs that included case enrollment and clinical data abstraction. Patient baseline characteristics, including age, gender, anthropometric indices (height, weight), primary diagnosis, and Pediatric Critical Illness Score (PCIS), were systematically documented within 48 h of PICU admission. Subsequently, longitudinal clinical surveillance was implemented, with synchronized collection of respiratory function indicators, hemodynamic parameters, and pulmonary ultrasonography findings performed within 48–72 h after initiating mechanical ventilation. Outcome metrics, including weaning success rates, ventilator-free days, and ICU length of stay (LOS), were longitudinally monitored until PICU discharge. To ensure data accuracy, a dual-researcher verification protocol was employed for independent data entry and cross-validation, with discrepancies resolved through consensus review by a senior intensivist.

### 2.5 Statistical analysis

The study population was randomly partitioned using The R Project for Statistical Computing (version 3.1.2), with 70% allocated to the modeling cohort and 30% to the validation cohort. Statistical analyses were conducted using SPSS Statistics (version 24.0). Continuous variables were analyzed via independent t-tests for normally distributed data (assessed by the Shapiro–Wilk test) or reported as medians with interquartile ranges (IQR) and analyzed using Mann–Whitney U tests for non-normally distributed data. Categorical variables were expressed as frequencies (percentages), with intergroup comparisons performed using Pearson's *χ*² test or Fisher's exact test, as appropriate.

Univariate logistic regression analysis identified candidate variables (retention threshold: *P* ≤ 0.20) for subsequent inclusion in multivariate binary logistic regression modeling. Variables achieving statistical significance (*P* < 0.05) in the multivariate analysis were retained in the final predictive model, with coefficients expressed as adjusted odds ratios (ORs) and 95% confidence intervals (CIs).

A prognostic nomogram was constructed using R (version 3.6.1), with internal validation performed via 1,000 bootstrap resamples to assess model accuracy and goodness-of-fit. Model discrimination was evaluated through receiver operating characteristic (ROC) curve analysis, quantified by the area under the curve (AUC), sensitivity, and specificity (*P* < 0.05 deemed statistically significant). Calibration performance was assessed using Kolmogorov–Smirnov plots, with concordance between predicted probabilities and ideal probabilities interpreted as evidence of model robustness. ROC analyses were independently validated by a institutional statistician using R 3.1.2, confirming cutoff stability via Youden's index.

## 3 Results

### 3.1 Incidence and clinical characteristics of children with difficult weaning

**Figure 1:**
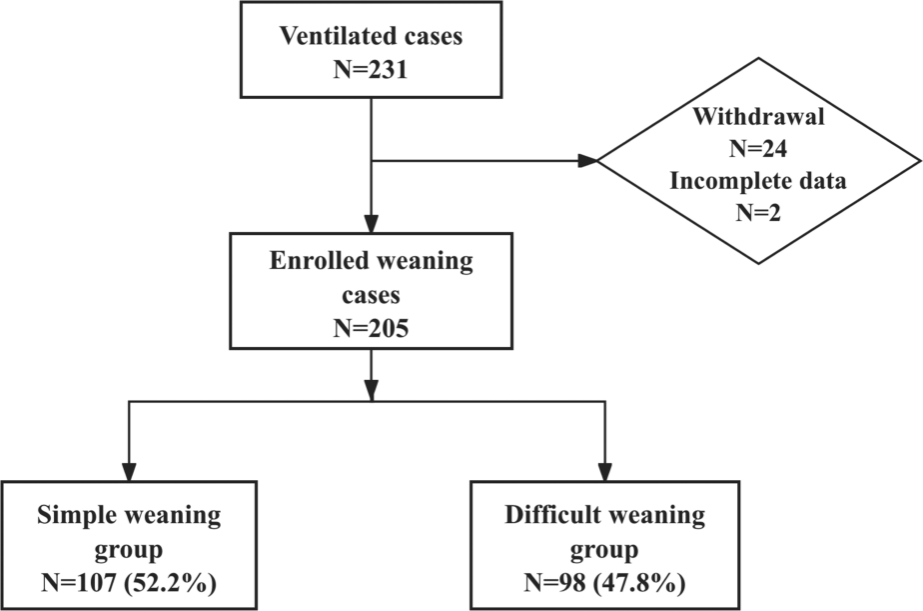
Flowchart.

A total of 231 pediatric patients met the inclusion criteria for this phase of the study. After applying stringent exclusion criteria, 26 cases were excluded due to mortality without weaning attempts (*n* = 17), treatment discontinuation (*n* = 3), unplanned extubation (*n* = 4), and incomplete data (*n* = 2), leaving 205 analyzable cases (Figure 1). These patients were stratified into two cohorts based on weaning outcomes: simple weaning group (*n* = 107, 52.2%) and the difficult weaning group (*n* = 98, 47.8%), indicating a difficult weaning incidence of 47.8%. In the difficult weaning group, 16 cases (7.8%) experienced extubation failure, and the median duration of intubation was 15 days, the median ICU length of stay (LOS) was 25 days. Patients in the difficult weaning group demonstrated significantly prolonged clinical trajectories compared to those in the simple weaning group (all *P* < 0.05). The detailed results of the analysis on incidence and clinical characteristics are presented in Table 2.

**Table 2:** Demographic characteristics and clinical outcomes in simple and difficult weaning groups.

Clinical characteristics and outcomes	Simple weaning group *N* = 107	Difficult weaning roup *N* = 98	*χ* ^2^ */Z*	*P* value
Gender, *n* (%)				
Male	50 (24.4)	47 (22.9)	*χ*^2^ = 0.031	0.860
Female	57 (27.8)	51 (24.9)		
Age group, years, *n* (%)			*χ*^2^ = 0.928	0.819
Infant (1–12 months)	53 (25.9)	55 (26.8)		
Toddler (1–3 years)	28 (13.7)	23 (11.2)		
School-aged (3.1–12 years)	16 (7.8)	12 (5.9)		
Adolescent (12.1–18 years)	10 (4.9)	8 (3.9)		
Diagnostic category, *n* (%)			*χ*^2^ = 9.341	0.096
Respiratory diseases	35 (17.1)	25 (12.2)		
Sepsis	10 (4.9)	17 (8.3)		
Cardiovascular diseases	34 (16.6)	24 (11.7)		
Hematological diseases	16 (7.8)	17 (8.3)		
Neurological diseases	5 (2.4)	12 (5.9)		
Other diseases	7 (3.4)	3 (1.5)		
Extubation outcome, *n* (%)			*χ*^2^ = 18.948^a^	<0.001
Successful extubation	107 (52.5)	82 (40.0)		
Unsuccessful extubation	0 (0.0)	16 (7.8)		
Endotracheal intubation duration, day	7 (6, 9)	15 (12, 21)	Z = −9.702^b^	<0.001
ICU length of stay, day	12 (9, 14)	25 (12, 34)	Z = −8.816^b^	<0.001
Timing of weaning initiation, day	6 (5, 8)	8 (6, 10)	Z = −4.333^b^	<0.001
Weaning Duration, day	1 (0.5, 1)	7(5, 10)	Z = −10.874^b^	<0.001

^a^ Pearson chi square.

^b^ Kruskal Wallis nonparametric test.

### 3.2 Characteristics and univariate analysis of patients with different weaning outcomes in the modeling cohort

A total of 143 cases (70%) were randomly allocated to the modeling group using R statistical software (version 3.6.1) and subsequently stratified into the simple weaning group and the difficult weaning group based on weaning outcomes. Univariate comparative analysis revealed statistically significant differences between groups in terms of serum albumin levels, PCIS (Pediatric Critical Illness Score), LUS (Lung Ultrasound Score), and SaO_2_ (arterial oxygen saturation) (*P* < 0.05). Difficult weaning patients had significantly higher LUS scores (*P* < 0.001) and lower PCIS (*P* < 0.001), indicating severe pulmonary dysfunction impedes liberation. Comprehensive analytical results are presented in Table 3.

**Table 3:** Comparison of patients’ characteristics in the modeling cohort according to weaning outcome.

Characteristics	Simple weaning group *N* = 77	Difficult weaning group *N* = 66	*χ^2^/Z*	*P* value
Gender, *n* (%)			*χ*^2^ = 0.322^a^	0.570
Male	36 (25.2)	34 (23.8)		
Female	41 (28.7)	32 (22.4)		
Age group, years, *n* (%)			*χ*^2^ = 0.316^a^	0.957
Infant (1–12 months)	42 (29.4)	38 (26.6)		
Toddler (1–3 years)	18 (12.6)	13 (9.1)		
School-aged (3.1–12 years)	14 (9.8)	12 (8.4)		
Adolescent (12.1–18 years)	3 (2.1)	3 (2.1)		
Diagnostic category, *n* (%)			*χ*^2^ = 9.444^a^	0.093
Respiratory diseases	24 (16.8)	15 (10.5)		
Sepsis	8 (5.6)	13 (9.1)		
Cardiovascular diseases	25 (17.5)	16 (11.2)		
Hematological diseases	9 (6.3)	13 (9.1)		
Neurological diseases	5 (3.5)	8 (5.6)		
Other diseases	6 (4.2)	1 (0.7)		
Relevant indicators of mechanical ventilation within 48–72 h				
Nutritional status, *n* (%)			*χ*^2^ = 0.445^a^	0.558
Normal nutrition status	57 (39.9)	52 (36.4)		
Malnutrition	20 (14.0)	14 (9.8)		
ALB	36.2 (33.1, 40.2)	34.8 (31.9, 37.3)	Z = −2.023^b^	0.043
Use of vasoactive drugs, *n* (%)			*χ*^2^ = 1.215^a^	0.327
Yes	8 (5.6)	11 (7.7)		
No	69 (48.2)	55 (38.5)		
Use of muscle relaxant, *n* (%)			*χ*^2^ = 0.782^a^	0.377
Yes	5 (3.5)	7 (4.9)		
No	72 (50.3)	59 (41.3)		
Muscle relaxant duration, day	2 (1, 6)	4 (1, 7)	Z = −0.355^b^	0.722
Percentage fluid overload (>10%), *n* (%)			χ^2^ = 2.122	0.115
Yes	6 (4.2)	11 (7.7)		
No	71 (49.6)	55 (38.5)		
PCIS	74 (74, 76)	72 (70, 74)	Z = −5.733^b^	<0.001
RASS	−1 (−2, 1)	−2 (−3, 1)	Z = −1.144^b^	0.254
LUS	19 (18, 20)	21 (20, 22)	Z = −6.374^b^	<0.001
rSO_2_	77 (76, 87)	75 (74, 86)	Z = −0.913^b^	0.361
SaO_2_	97.4 (95.7, 98.6)	96.8 (94.3, 98.2)	Z = −2.017^b^	0.044
PaCO_2_	40.1 (37.2, 44.1)	41.3 (38.1, 45.6)	Z = −0.478^b^	0.633
PH	7.39 (7.35, 7.42)	7.40 (7.34, 7.44)	Z = −1.041^b^	0.298
PaO_2_/FiO_2_ ratio	186.3 (170.8, 203.3)	178.8 (156.4, 205.4)	Z = −1.432^b^	0.152
HB	98.0 (89.0, 106.5)	94.0 (85.3, 104.5)	Z = −1.697^b^	0.090
Lac	1.3 (0.9, 1.7)	1.6 (1.0, 1.8)	Z = −0.837^b^	0.404

Percentage fluid overload = [(Total Fluid Intake in Liters−Total Fluid Output in Liters)/Admission Weight in Kilograms] × 100%; PCIS, pediatric critical illness score; RASS, richmond agitation-sedation scale; LUS, lung ultrasound score; rSO_2_, regional cerebral oxygen saturation; SaO_2_, oxyhemoglobin saturation; PaCO_2_, arterial carbon dioxide tension; ALB, serum albumin; HB, hemoglobin concentration; Lac, lactate levels.

^a^ Pearson chi square.

^b^ Kruskal Wallis non parametric test.

### 3.3 Risk factor analysis for difficult weaning in pediatric patients at 48–72 h of mechanical ventilation

Multivariable logistic regression analysis was conducted using variables with *P* ≤ 0.2 from univariate analysis as screening criteria. Based on the results presented in Table 3, seven variables—disease diagnosis, PCIS (Pediatric Critical Illness Score), LUS (Lung Ultrasound Score), SaO₂ (arterial oxygen saturation), P/F ratio (oxygenation index), hemoglobin (Hb), and serum albumin (ALB)—were included in the regression model, with difficult weaning outcome as the dependent variable. The final model identified two independent risk factors with statistical significance (*P* < 0.05): PCIS (adjusted odds ratio [aOR] = 0.748, 95% confidence interval [CI]: 0.639–0.875, *P* = 0.001), indicating that lower severity scores were associated with a higher risk of weaning failure; and LUS (aOR = 2.316, 95% CI: 1.668–3.216, *P* < 0.001), demonstrating that elevated LUS values significantly predicted difficult liberation. These findings suggest that reduced physiological reserve (as reflected by lower PCIS) and impaired pulmonary aeration (as quantified by higher LUS) independently contribute to weaning failure. Comprehensive regression outputs are detailed in Table 4.

**Table 4:** Stepwise logistic regression analysis of risk factors associated with difficult weaning in pediatric patients.

Variable	B	SE	Wald value	*P* value	Odds ratio	95%CI
Diagnostic category	−0.025	0.158	0.024	0.877	0.976	(0.715–1.331)
Respiratory diseases	–					
Sepsis	0.956	0.557	1.715	0.086	2.600	(0.888–8.023)
Cardiovascular diseases	0.024	0.459	0.052	0.959	1.024	(0.415–2.533)
Hematological diseases	0.838	0.544	1.539	0.124	2.311	(0.805–6.908)
Neurological diseases	0.940	0.658	1.428	0.153	2.560	(0.719–9.899)
Other diseases	−1.322	1.129	−1.171	0.242	0.267	(0.013–1.778)
LUS	0.829	0.167	25.155	<0.001	2.316	(1.668–3.216)
PCIS	−0.278	0.08	13.096	0.001	0.748	(0.639–0.875)
SaO_2_	−0.172	0.099	3.019	0.082	0.842	(0.748–1.074)
PaO_2_/FiO_2_ ratio	0.001	0.008	0.017	0.897	1.001	(0.984–1.013)
HB	0.001	0.017	0.004	0.952	1.001	(0.965–1.029)
ALB	−0.061	0.054	1.285	0.257	0.941	(0.845–1.034)

PCIS, pediatric critical illness score; LUS, lung ultrasound score; SaO_2_, oxyhemoglobin saturation; ALB, serum albumin; HB, hemoglobin concentration.

### 3.4 Development and validation of a nomogram model for early prediction of difficult weaning in pediatric patients

**Figure 2:**
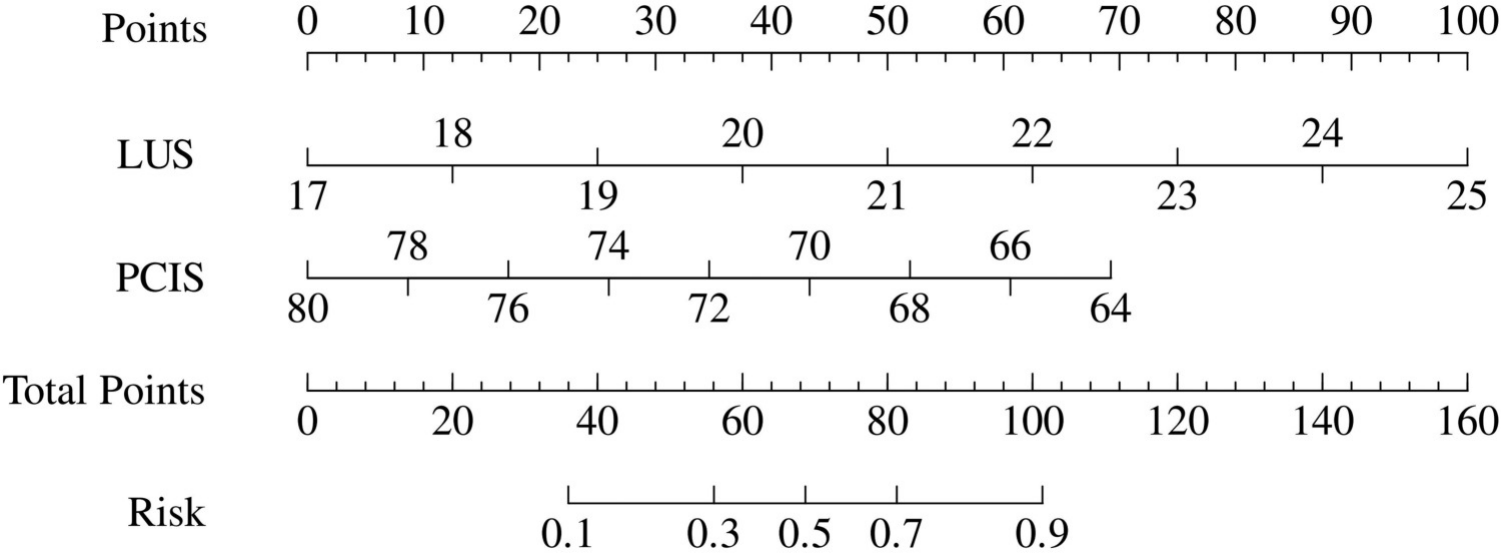
Risk nomogram model conducted by logistic regression (LUS, lung ultrasound score; PCIS, pediatric critical illness score).

Based on the aforementioned statistical findings, a prognostic nomogram model was developed to predict the early risk of difficult weaning in mechanically ventilated pediatric patients (Figure 2). The model integrates two independent predictors identified through multivariable logistic regression analysis: critical illness severity score (PCIS) and lung ultrasound score (LUS). Input variables were weighted according to their regression coefficients, with output parameters including risk factor-specific points, total score, and predicted probability of weaning failure.

**Figure 3:**
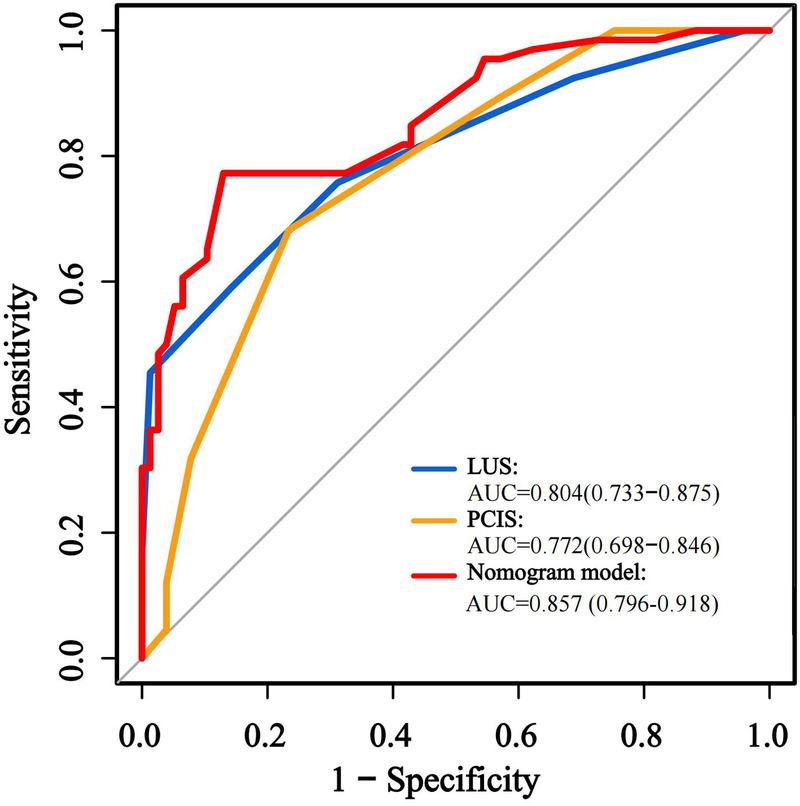
ROC curves of the modeling cohort.

The predictive performance of the nomogram was evaluated against individual risk factors using receiver operating characteristic (ROC) curve analysis (Figure 3; Table 5). The nomogram demonstrated superior discriminative capacity, achieving an area under the curve (AUC) of 0.857 (95% CI: 0.79–0.92, *P* < 0.001), significantly outperforming the predictive efficacy of either isolated PCIS (AUC = 0.772, 95% CI: 0.69–0.84) or LUS (AUC = 0.804, 95% CI: 0.73–0.87).

**Table 5:** Efficacy and validation analysis of the prediction models and various indicators for difficult weaning in pediatric patients.

Index	Cut off value	Sensitivity (95% CI)	Specificity (95% CI)	AUC (95% CI)	Youden index	PPV (95% CI)	NPV (95% CI)	*P* value
LUS	≥20	75.8 (63.6–85.5)	68.8 (57.3–78.9)	0.804 (0.73–0.87)	0.45	78.0 (66.4–86.4)	76.8 (67.8–83.9)	<0.00
PCIS	≤72	68.2 (55.6–79.1)	76.6 (65.6–85.5)	0.772 (0.69–0.84)	0.45	71.4 (61.8–79.5)	73.7 (65.9–80.3)	<0.00
Nomogram in modeling cohort	0.55	87.0 (79.5–94.5)	77.3 (67.2–87.4)	0.857 (0.79–0.91)	0.63	81.7 (73.3–90.1)	83.6 (74.3–92.9)	<0.00
Nomogram in validation cohort	0.55	84.3 (70.0–96.7)	76.7 (59.2–82.1)	0.854 (0.76–0.95)	0.61	79.4 (64.4–84.5)	82.1 (65.6–95.9)	<0.00

LUS, lung ultrasound score; PCIS, pediatric critical illness score.

**Figure 4:**
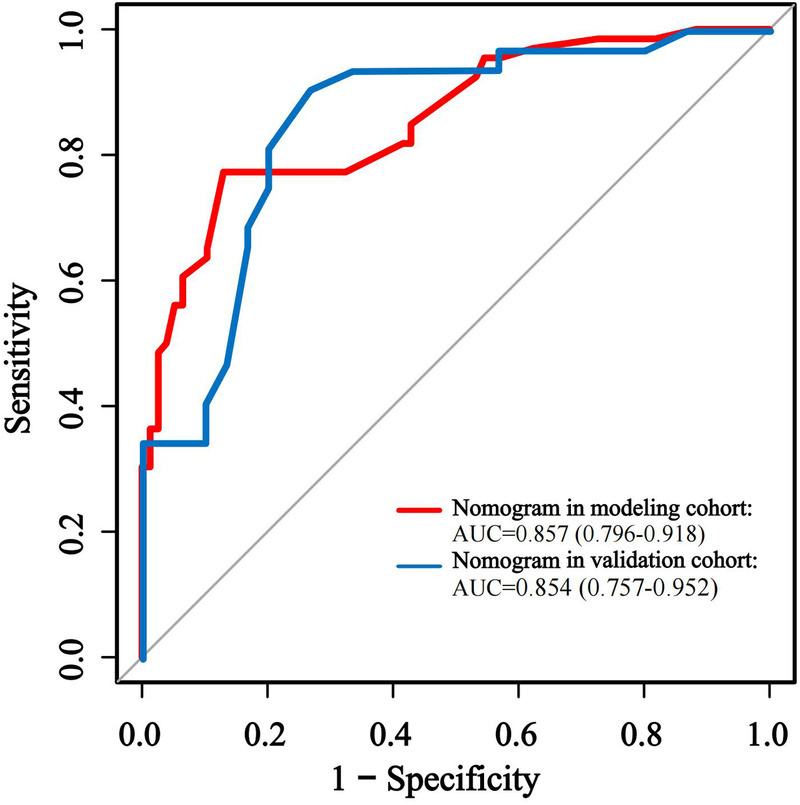
ROC curves of the validation cohort.

The effectiveness of the developed nomogram model was evaluated in the rest validation cohort comprising 62 cases (30%). Through the use of receiver operating characteristic (ROC) curve analysis (Figure 4; Table 5), the model demonstrated robust discriminative performance with an area under the curve (AUC) of 0.854 (95% CI: 0.76–0.95, *P* < 0.001), showing no statistically significant difference compared to its performance in the modeling cohort (DeLong's test: Z = 1.460, *P* = 0.144).

**Figure 5:**
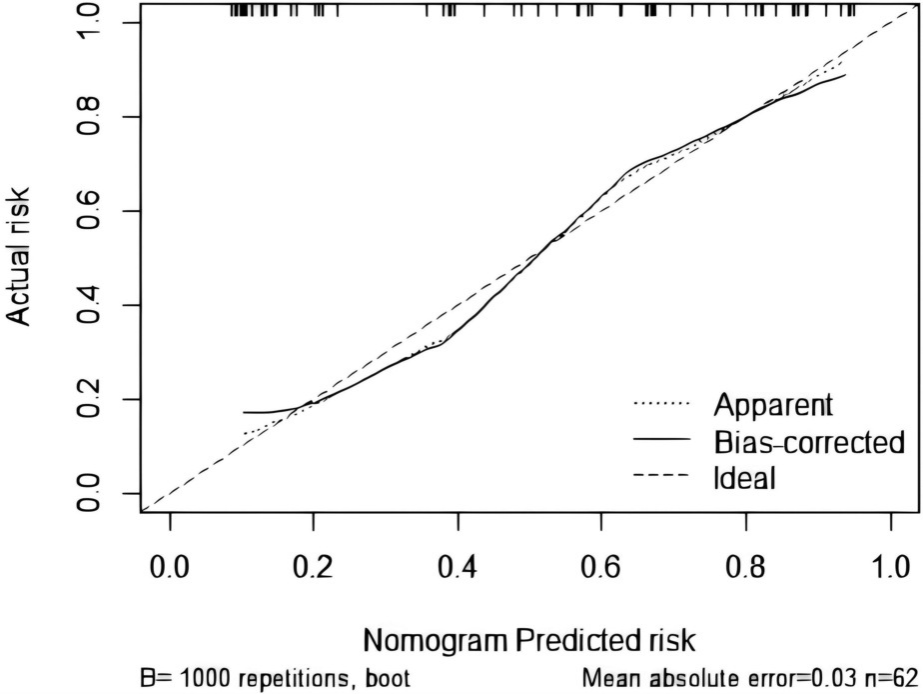
Kolmogorov–Smirnov calibration curve.

The goodness-of-fit analysis of the model was conducted using 1,000 bootstrap resamples via R statistical software, followed by Kolmogorov–Smirnov calibration curve analysis (Figure 5). The bias-corrected curve, derived from 1,000 bootstrap iterations, exhibited excellent concordance with the apparent curve, thereby indicating negligible model variability and robust reliability.

## 4 Discussion

Difficult weaning affects 37.2%–45% of critically ill children and critically impacts outcomes (3, 4). It significantly prolongs mechanical ventilation duration and hospital stays while increasing risks of extubation failure and mortality (19, 20). A Lancet multinational cohort study revealed that 15% of difficult-weaning patients experienced extubation failure, with 78% progressing to in-hospital death (21). Current guidelines recommend protocolized weaning with spontaneous breathing trials (SBTs) for safe adult liberation (1, 6). However, children face greater liberation challenges due to immature physiological responses and multifactorial influences (4). Pediatric studies remain limited in scope and statistical power for generalizable conclusions, while persistent controversies over optimal SBT protocols—particularly regarding duration and termination criteria—hinder standardized pediatric frameworks (22). SBTs primarily rely on basic metrics (respiratory rate, heart rate, blood gases) that difficultly reflect respiratory reserve or predict post-extubation outcomes in children (23). Although SBTs reduce ventilation duration, they fail to lower high difficult-weaning rates (47.8% in this study, with 7.8% progressing to failure; *P* < 0.05 vs. simple weaning), consistent with global epidemiology (13, 24). These findings highlight fundamental limitations of conventional SBTs for pediatric liberation.

Pediatric weaning assessment presents unique challenges that distinguish it from adult practice. Greater physiological heterogeneity in children leads to less predictable liberation outcomes, influenced by a combination of developmental, pathological, and iatrogenic factors (4, 22, 25, 26). Predictors validated in adult populations—such as the rapid shallow breathing index (RSBI), P/F ratio, dynamic compliance (Cydn), CROP index, serum albumin, and hemoglobin levels—show inconsistent reliability and limited utility in guiding pediatric extubation decisions. The Burns Wean Assessment Program (BWAP), which incorporates 26 multidimensional parameters for predicting adult liberation outcomes, demonstrates limited applicability in pediatric settings (27, 28). Current pediatric ventilation guidelines recognize significant evidence gaps in weaning protocols, emphasizing the lack of high-quality research to define optimal strategies or reliable predictors specifically for children. This knowledge deficit may delay weaning initiation and impair the timely identification of modifiable risk factors during critical phases (5, 25, 26).

Our study aims to develop an accurate predictive model to help clinicians identify modifiable risks early during mechanical ventilation, thereby optimizing practice and improving liberation success and weaning quality. We innovatively identify early predictors of difficult pediatric weaning and establish a nomogram integrating quantitative Lung Ultrasound Score (LUS) with the Pediatric Critical Illness Score (PCIS) for real-time risk monitoring. Results show that lower PCIS values indicate reduced physiological reserve, aligning with known links between critical illness severity and extubation difficulty (29). PCIS significantly predicted difficult weaning (AUC 0.772, 95% CI: 0.692–0.852; sensitivity 68.2%, specificity 76.6%), corroborating prior studies (30). Complementing PCIS, LUS provides radiation-free bedside assessment of lung aeration, offering advantages over static imaging like CT or x-ray (8, 10). Elevated LUS scores reflect impaired pulmonary aeration and increased lung injury severity (31). Early LUS evaluation showed significantly higher scores in difficult-weaning patients than in simple-weaning patients, suggesting more severe parenchymal injury, reduced ventilation, or fluid overload. As a standalone predictor, LUS effectively identified subclinical impairments and predicted outcomes (AUC 0.804, 95% CI: 0.729–0.879; sensitivity 75.8%, specificity 68.8%). Compared to diaphragm-focused models (30, 32) or traditional parameters like RSBI/PF ratio—which have limited pediatric utility (25)—our integrated nomogram allows earlier, more comprehensive risk stratification, supported by superior discrimination (AUC 0.874) and stable calibration. This nomogram provides clinicians intuitive visual tools to assess weaning difficulty severity, identify modifiable risks, and guide individualized management. The model underwent rigorous internal validation (1,000 bootstrap resamples) and external calibration, demonstrating strong discrimination, accurate calibration, and temporal stability.

While this study leverages LUS's visual quantification to address limitations of conventional pediatric extubation predictors and improve model discrimination, it has several notable limitations. Children with neuromuscular disorders or congenital airway malformations—who are at increased risk of extubation failure due to extrapulmonary factors (23)—were excluded, as LUS provides limited diagnostic value in these subgroups. Furthermore, although the model demonstrates robust overall accuracy, it may not guarantee precision in individual cases. The Kolmogorov–Smirnov (K-S) calibration curve reveals systematic deviations of predicted probabilities from the ideal reference line, indicating potential miscalibration and highlighting the need for further model refinement through larger, multicenter studies.

## 5 Conclusion

Difficult weaning represents a significant clinical challenge in Pediatric Intensive Care Units, with strong associations with adverse outcomes. Therefore, clinicians should focus not only on the final weaning outcome but also on comprehensive monitoring of the entire weaning process. Our nomogram prediction model functions as a decision-support tool for early risk stratification, enabling proactive and systematic identification of modifiable risk factors for extubation failure and facilitating timely assessment of weaning readiness. These findings underscore the value of integrated predictive models in bridging physiological monitoring with clinical decision-making, ultimately improving the delivery of precision care in pediatric critical care settings.

## Data Availability

The original contributions presented in the study are included in the article/Supplementary Material, further inquiries can be directed to the corresponding authors.
